# Digitized smart surveillance and micromanagement using information technology for malaria elimination in Mangaluru, India: an analysis of five-year post-digitization data

**DOI:** 10.1186/s12936-021-03656-8

**Published:** 2021-03-08

**Authors:** B. Shantharam Baliga, Shrikala Baliga, Animesh Jain, Naveen Kulal, Manu Kumar, Naren Koduvattat, B. G. Prakash Kumar, Arun Kumar, Susanta K. Ghosh

**Affiliations:** 1 Kasturba Medical College Mangalore, Manipal Academy of Higher Education, Manipal, Karnataka India; 2 Manipal Center for Infectious Diseases, Prasanna School of Public Health, Manipal Academy of Higher Education, Manipal, Karnataka India; 3 Department of Public Health, Dakshina Kannada District, Mangalore, Karnataka 575001 India; 4 Officer On Special Duty, Chief Minister’s Secretariat Bengaluru, Bangalore, Karnataka 560001 India; 5 I-Point Consulting, Punja Arcade, Lalbagh, Mangalore, Karnataka 575003 India; 6 Directorate of Health and Family Welfare Services, Government of Karnataka, Bangalore, Karnataka 560009 India; 7 General Hospital, Shikaripura, Karnataka 577427 India; 8 ICMR-National Institute of Malaria Research (Field Unit), Nirmal Bhawan, ICMR Campus, Poojanahalli, Kannamangla Post, Devanahalli Taluk, Bangalore, Karnataka 562110 India; 9 Manipal Academy of Higher Education, Manipal, Karnataka 576104 India

**Keywords:** Malaria, Digitization, GIS, TAB, Smart surveillance, Micromanagement, Malaria elimination, Information technology, Mangaluru

## Abstract

**Background:**

Malaria control system (MCS), an Information technology (IT)-driven surveillance and monitoring intervention is being adopted for elimination of malaria in Mangaluru city, Karnataka, India since October 2015. This has facilitated ‘smart surveillance’ followed by required field response within a timeline. The system facilitated data collection of individual case, data driven mapping and strategies for malaria elimination programme. This paper aims to present the analysis of post-digitization data of 5 years, discuss the current operational functionalities of MCS and its impact on the malaria incidence.

**Methods:**

IT system developed for robust malaria surveillance and field response is being continued in the sixth year. Protocol for surveillance control was followed as per the national programme guidelines mentioned in an earlier publication. Secondary data from the malaria control system was collated and analysed. Incidence of malaria, active surveillance, malariogenic conditions and its management, malariometric indices, shrinking malaria maps were also analysed.

**Results:**

Smart surveillance and subsequent response for control was sustained and performance improved in five years with participation of all stakeholders. Overall malaria incidence significantly reduced by 83% at the end of 5 years when compared with year of digitization (DY) (*p* < 0.001). Early reporting of new cases (within 48 h) was near total followed by complete treatment and vector control. Slide positivity rate (SPR) decreased from 10.36 (DY) to 6.5 (PDY 5). Annual parasite incidence (API) decreased from 16.17 (DY) to 2.64 (PDY 5). There was a negative correlation between contact smears and incidence of malaria. Five-year data analyses indicated declining trends in overall malaria incidence and correlation between closures by 14 days. The best impact on reduction in incidence of malaria was recorded in the pre-monsoon months (~ 85%) compared to lower impact in July–August months (~ 40%).

**Conclusion:**

MCS helped to micromanage control activities, such as robust reporting, incidence-centric active surveillance, early and complete treatment, documentation of full treatment of each malaria patient, targeted mosquito control measures in houses surrounding reported cases. The learnings and analytical output from the data helped to modify strategies for control of both disease and the vector, heralding the city into the elimination stage.

## Background

Globally malaria is still a major public health problem although the scenario has changed a little since 2015 [1–4]. The global incidence of malaria has reduced from 71 to 57 per 1000 population at risk [1]. The challenges faced are operational, health system deficiencies and poor management systems [2, 5]. Information technology (IT) system is critical for malaria elimination to improve surveillance, complete case reporting, data analysis that lead to timely responses in the field leading to robust and responsive surveillance [6]. Available global IT systems have been evaluated with regards to the structure of the system, data captured, output, strengths and challenges [6]. These authors have identified the inability to capture private health sector data, nil documentation of field response, failure to map the cases, difficulty in tracking migrant workers, failure to capture time of reporting, inability to capture data in real time, and non-integration with mobile technology, as challenges of using IT systems. Malaria surveillance data available through the routine malaria information system (MIS) that was used did not provide the much needed information on severe malaria cases, since a large number of patients seek health care from the private sectors, and these did not figure in the programme data [6, 7].

Mangaluru (Mangalore) is a coastal city in Karnataka of southwestern India. The city has administrative units designated as wards, and 60 such wards constitute the city limits [8]. Malaria has been endemic in Mangaluru for three decades [1–3]. Malaria control measures were being carried out as per the guidelines of National Vector Borne Disease Control Programme (NVBDCP); however, desired results were not observed till 2014. To address the deficiency of the existing systems and to improve the performance of control strategies, a new IT system namely Malaria Control System (MCS) was launched in October 2015 in Mangaluru, and is operational till date [9]. This IT system was introduced to capture data and build capacity of existing programme in the entire city.

MCS consists of an innovative handheld, Android-based geographical information system (GIS)-tagged tablets (TABs) device, and a web-based incident reporting system. The system ensures `smart surveillance’ coupled with field response and data collection for analysis to design local strategies for malaria elimination. MCS was introduced as a programme management system, and as an intervention to assist effective management of malaria control programme by digitizing the reporting of newly diagnosed malaria cases for treatment, tracking and closure of cases after complete treatment of each malaria patient. Malaria control software is being used for the sixth consecutive year and cases are reported by all the health care providers and stakeholders including the private sectors. Field activities for control and closure of cases and source elimination of breeding habitats are carried out based on the inputs into the software. Routine monitoring and strict vigils were put in place on the ongoing newly introduced surveillance system using GIS-tagged TABs. A previous article has described the design and implementation of this IT system protocol and presented initial secondary data analyses to determine the impacts in 2-year post-digitization [9].

In the post-digitization years, it was easier to access and retrieve the data. Hence routine real time monitoring and analyses of malaria indices in all the wards covering the entire city limits was possible. The administrators were able to identify high-risk areas periodically to carry out necessary additional anti-malarial activities.

This paper aims to present the analyses of five-year post-digitization data, discuss the current operational functionalities of MCS and its impact on the malaria incidence.

## Methods

MCS is being continued as a management and monitoring tool in the city of Mangaluru since October 2015 [9]. Early reporting within 24 to 48 h followed by field response in next 24 to 48 h along with anti-mosquito measures were carried out as per the NVBDCP guidelines. This data available on the IT system was translated onto excel sheets and were analysed for taking appropriate decisions and amendments in the action plan. Secondary analyses of five-year data were also carried out.

Malaria cases reported in the city were analysed based on the type of health facilities from where the patients sought health care services and its reporting. These health care facilities were categorized as private health facilities, and public health facilities. Private health facilities included all the hospitals, nursing homes and diagnostic laboratories. Public Health facilities included surveillance team of district vector borne disease control office (DVBDCO), government-run hospitals, urban health centres and malaria clinics.

Each malaria case was analysed based on reporting time, complete treatment and closure of the cases subsequent to follow-up smear examination for clearance of parasites, and closure of cases within day 14 and also within 30 days in some cases. Closure time is considered as 14 days to complete radical treatment with primaquine for *Plasmodium vivax* cases to prevent relapse as per the recommendation of NVBDCP [10]. Anti-vector responses in the field were also analysed.

Factual reporting with regards to administrative decisions, hurdles in the implementation of anti-malarial activities, how these problems were addressed, and their effects on the malaria control were noted with proper documentation. The malaria indices were additionally analysed to assess the impact of intervention.

### Definitions

Smart surveillance was initiated from October 2014 to September 2015, and was considered as digitization year (DY) [9]; October 2015 to September 2016 as post-digitization year 1 (PDY 1); October 2016 to September 2017 as post-digitization year 2 (PDY 2) [9]; October 2017 to September 2018 as post-digitization year 3 (PDY 3); October 2018 to September 2019 as post-digitization year 4 (PDY 4) and October 2019 to September 2020 as post-digitization 5 (PDY 5).

### Statistical analysis

Closure and closure time of each positive case and vector interventions were analysed. Community visits, contact smears during active surveillance around reported case (ASARC), vector control activities were analysed along with malaria indices such as Annual Blood Examination Rate (ABER), Slide Positivity Rate (SPR), Slide Falciparum Rate (SFR) and Annual Parasite Incidence (API). Monthly trends of malaria at each level were also plotted in relation to closure of cases. Fischer *F* test was applied to find the significance in reduction of malaria cases. Time series analysis was done for plotting the trends of closure rate of cases against the incidence of cases of malaria. Bonferroni *t* test was used to test the statistical significance of inter time interval. A *p* value of < 0.05 was considered statistically significant.

## Results

Monthly incidence of malaria for the past 6 years and the cumulative reduction in incidence in urban limits of Mangaluru is depicted in Table 1. Gradual reduction of overall incidence of malaria continued throughout five-year post-digitization (PDY 5) with an overall cumulative reduction by 83% (range -64% to -92%) as compared to digitization year (DY).

**Table 1:** Monthly incidence of malaria in Mangaluru city

Month	2013–14	2014–15 DY	2015–16 PDY 1^a^	2016–17 PDY 2	2017–18 PDY 3	2018–19 PDY 4	2019–20 PDY 5	Cumulative reduction (%) (PDY 5)
October	479	1064	929	717	776	398	357	− 66
November	465	1278	1116	631	750	458	190	− 64
December	454	1103	1348	468	728	412	209	− 81
January	532	1101	1068	403	438	342	145	− 87
February	471	554	662	305	281	182	87	− 84
March	477	521	400	305	329	180	54	− 90
April	617	528	475	405	294	117	79	− 85
May	1004	715	449	374	384	106	64	− 91
June	1159	1065	1142	656	741	166	81	− 92
July	1526	971	2084	1174	1003	581	158	− 84
August	1062	848	1952	1325	837	514	230	− 78
September	1421	1224	989	874	549	294	159	− 87
Total	9667	10,972	12,641	7637	7110	3750	1813	− 83

^a^Some diagnostic centres reported cases directly to malaria control cell. Yearly reduction is found to be highly significant. F value 17.737, *p* value < 0.00. Using the Bonferroni *t* test, the reduction in incidence was statistically significant as seen from the *p* values for inter-time interval between various years; between PDY 1 and PDY 2 (*p* < 0.05); PDY 1 against PDY 3(*p* < 0.01); and between PDY 4 and PDY 5 (*p* < 0.001)

The maximum cumulative reduction of 91 to 92% in incidence was noted for the months of May and June and least 64 to 66% in the months of October and November soon after the monsoon season. The ward-level cumulative reduction in the incidence is also depicted in Table 2. The range of reduction of cumulative incidence is 45% (Court ward no. 40) to 98% (Maroli ward no. 37). The ward-level cumulative reduction in incidence of malaria from the PDY 1 to PDY 5 was significant (*p* < 0.001).

**Table 2:** Ward-level malaria cases in Mangaluru post-digitization and cumulative reduction

Ward	2015–16 PDY 1	2016–17 PDY 2	2017–18 PDY 3	2018–19 PDY 4	2019–20 PDY 5	Cumulative reduction (%) (PDY 5)
1-Surathkal-West	105	89	18	26	24	− 77
2-Surathkal-East	55	40	13	22	17	− 69
3-Katipalla-East	36	13	11	5	1	− 97
4-Katipalla-K’pura	17	16	13	11	2	− 88
5-Katipalla-North	22	10	14	10	6	− 72
6-Idya-East	49	126	68	26	3	− 93
7-Idya-West	25	15	17	7	3	− 88
8-Hosabettu	18	23	17	12	9	− 50
9-Kulai	0	20	32	8	8	− 60
10-Baikampady	77	44	40	22	8	− 91
11-Panambur	101	69	50	55	26	− 74
12-Panjimogaru	91	51	53	33	16	− 82
13-Kunjathbail –North	77	33	51	24	15	− 77
14-Marakada	56	17	54	28	7	− 88
15-Kunjathbail-South	60	37	61	20	3	− 95
16-Bangrakulur	74	73	88	52	13	− 82
17-Derebail-North	459	128	207	61	30	− 93
18-Kavoor	397	105	115	72	36	− 91
19-Pachanady	101	41	18	20	17	− 83
20-Thiruvail	31	14	0	8	2	− 93
21-Padavu-West	66	32	82	28	9	− 71
22-Kadri Padavu	291	209	135	82	37	− 87
23-Derebail-East	432	224	105	120	66	− 85
24-Derebail-South	109	254	277	51	17	− 84
25-Derebail-West	246	157	293	166	39	− 86
26-Derebail-North-East	212	123	201	159	25	− 88
27-Boloor	176	39	45	26	10	− 94
28-Mannagudda	346	110	170	31	15	− 97
29-Kambla	85	90	46	14	13	− 84
30-Kodialbail	186	134	140	65	53	− 71
31-Bejai	162	157	236	132	49	− 66
32-Kadri-North	131	75	55	107	20	− 84
33-Kadri-South	181	159	341	211	27	− 85
34-Shivabagh	113	74	93	47	13	− 88
35-Padavu-Central	120	124	124	56	16	− 87
36-Padavu-East	121	107	118	94	20	− 84
37-Maroli	107	65	58	15	2	− 98
38-Bendoor	205	113	74	29	13	− 94
39-Falnir	240	92	55	21	8	− 97
40-Court	293	322	438	180	160	− 45
41-Central Market	710	644	404	191	182	− 74
42-Dongarkeri	104	66	40	39	20	− 81
43-Kudroli	218	58	65	31	32	− 85
44-Bunder	799	496	400	248	155	− 81
45-Port	753	554	451	222	155	− 79
46-Cantonment	147	227	292	156	66	− 55
47-Milagrese	507	233	163	69	17	− 97
48-Kankanady Valencia	370	133	95	22	23	− 94
49-Kankanady	255	72	70	29	16	− 94
50-Alape-South	108	50	37	21	14	− 87
51-Alape-North	109	84	32	10	11	− 91
52-Kannur	43	29	36	18	9	− 79
53-Bajal	118	48	30	8	7	− 93
54-Jeppinamogaru	62	41	27	12	10	− 84
55-Attavara	307	181	101	54	14	− 95
56-Mangaladevi	418	174	181	64	26	− 96
57-Hoige Bazaar	278	412	205	112	38	− 86
58-Bolar	197	109	78	49	10	− 95
59-Jeppu	132	79	59	24	15	− 87
60-Bengre	446	323	294	193	104	− 77

In June 2018, comprehensive malaria elimination teams (CMETs) were formed to visit reported cases of malaria and to carry out sanitization of the area subsequent to administrative decision to utilize services of the designated multipurpose workers (MPWs) for non-malarial work. Consequent to functioning of CMETs resultant figures for malaria incidence in PDY 4 showed a marked reduction. It was noted that surveillance continued to improve with malaria cases being reported from all the hospitals and diagnostic centres of private as well as public health systems (Table 3). In the first year after digitization, private health care facilities contributed to nearly two-thirds (68%) of the total cases being reported; while the public health system contributed to nearly one-third (which included 18.6% by community public hospitals and 4.3% by malaria clinics). In the post-digitization phase, the contribution of total number of cases from the private hospitals kept steadily declining and reduced to 57% in the PDY 4. At the same time, the public health system, i.e. public hospitals, urban health centres as well as DVBDCO started contributing larger proportion of total number of cases. With the onset of COVID-19 pandemic, the private sector contribution was found to have increased again. The ASARC contributed to over 1.6% of malaria incidence during the fifth year, emphasizing the role played by it (Table 3).

**Table 3:** Type of health facilities and malarial case reports in Mangaluru city

	2015–16 PDY 1^a^	2016–17 PDY 2	2017–18 PDY 3	2018–19 PDY 4	2019–20 PDY 5
Total number of cases	11,757	7637	7110	3750	1813
District Vector borne Disease Control Office (DVBDCO)	571 (4.9%)	648 (8.5%)	593 (8.3%)	381 (10.2%)	67 (3.6%)
Public Hospitals	2184 (18.6%)	1157 (15.1%)	1406 (19.8%)	778 (20.7%)	347 (19.1%)
Urban health centers	329 (2.8%)	601 (7.9%)	811 (11.4%)	322 (8.9%)	139 (7.7%)
Active surveillance	123 (1.1%)	32 (0.4%)	55 (0.8%)	44 (1.2%)	32 (1.8%)
Malaria clinics	501 (4.3%)	327 (4.3%)	255 (3.58%)	89 (2.4%)	30 (1.6%)
Private Health facilities	8049 (68%)	4872 (63%)	4245 (56%)	2136 (57%)	1226 (68%)

^a^Cases directly reported to malaria control cell are not included

Table 4 depicts the number of cases for the last 5 years’ smears tested, contact smears taken and malariometric indices. There was a negative correlation between the ratio of contact smears of the total number of cases and number of positive cases detected by contact smears, albeit not statistically significant. The malarial indices were calculated for the pre-digitization year, digitization year and each of the five-year post-digitization periods. The SPR was seen decreasing steadily, while the average API came down to 2.64 in the PDY 5. The API, SPR, and SFR showed statistically significant changes (*p* < 0.001).

**Table 4:** Malaria incidence data, contact smears and malriometric indices in Mangaluru

	Pre-digitization	Digitization year (DY)	2015–16 PDY 1	2016–17 PDY 2	2017–18 PDY 3	2018–19 PDY 4	2019–20 PDY 5
Total malarial cases (no.)	8867	10,962	12,614	7637	7110	3750	1813
Number of smears collected	84,102	106,885	154,409	203,894	130,910	86,745	27,608
Number of contact smears Positive cases from ASARC	NA	NA	21,203 (123)	36,211 (32)	20,839 (55)	13,185 (44)	8656 (32)
Number of smears/incidence	9.48	9.75	12.24	26.68	18.37	23.18	16.75
Vivax malaria (% of total)	8092 (91)	10,196 (93)	11,277 (89)	6245 (82)	5633 (79)	3099 (82)	14 (82)
Falciparum Malaria (% of total)	775 (9)	766 (7)	1337 (11)	1395 (18)	1494 (21)	651 (18)	329 (18)
Chi-square for trend x^2^ = 679.63 *p* < 0.001							
ABER (%)	13.48	17.13	24.75	32.68	20.9	17.75	4.9
SPR (%)	11.15	10.36	8.17	3.74	5.4	4.3	6.56
SFR (%)	0.92	0.73	0.86	0.68	1.1	0.7	1.19
API (cases/1000 population)	15.51	16.17	18.42	12.24	11.4	5.4	2.64

*ABER* annual blood examination rate, *SPR* slide positivity rate; *SFR* slide falciparum rate, *API* annual parasite incidence

**Fig. 1:**
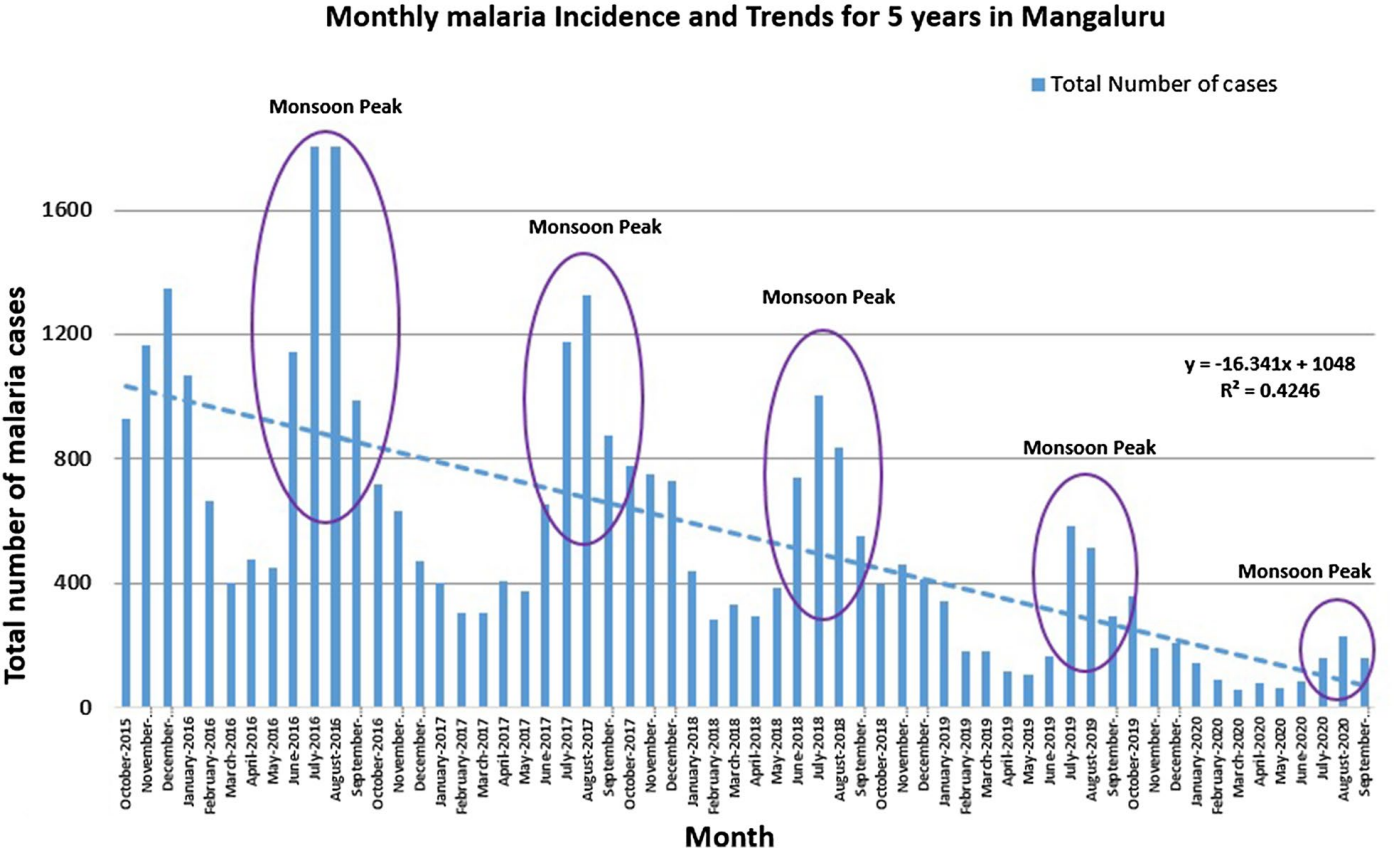
Monthly malaria Incidence and Trends for 5 years in Mangaluru

**Fig. 2:**
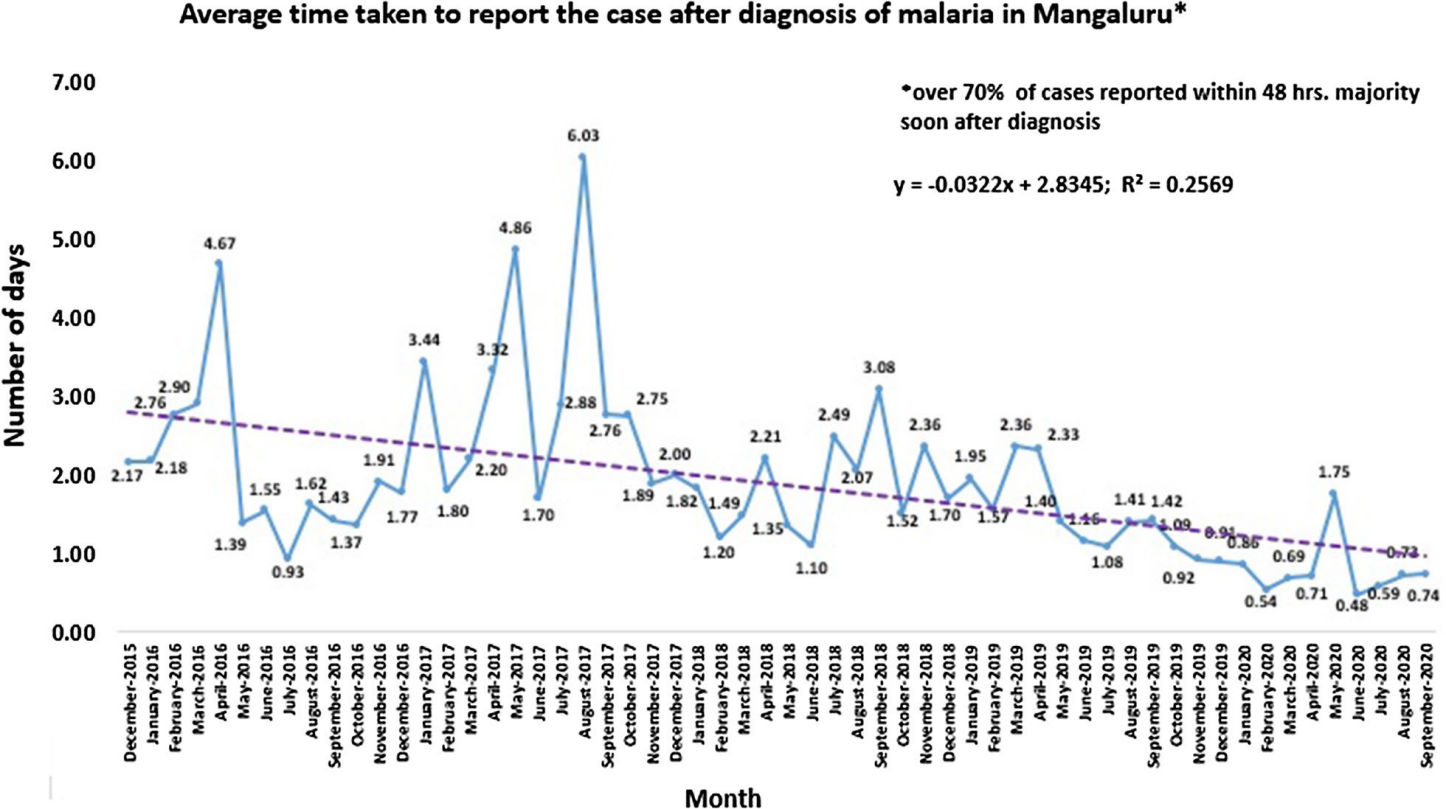
Average time taken to report the case after diagnosis of malaria in Mangaluru*

**Fig. 3:**
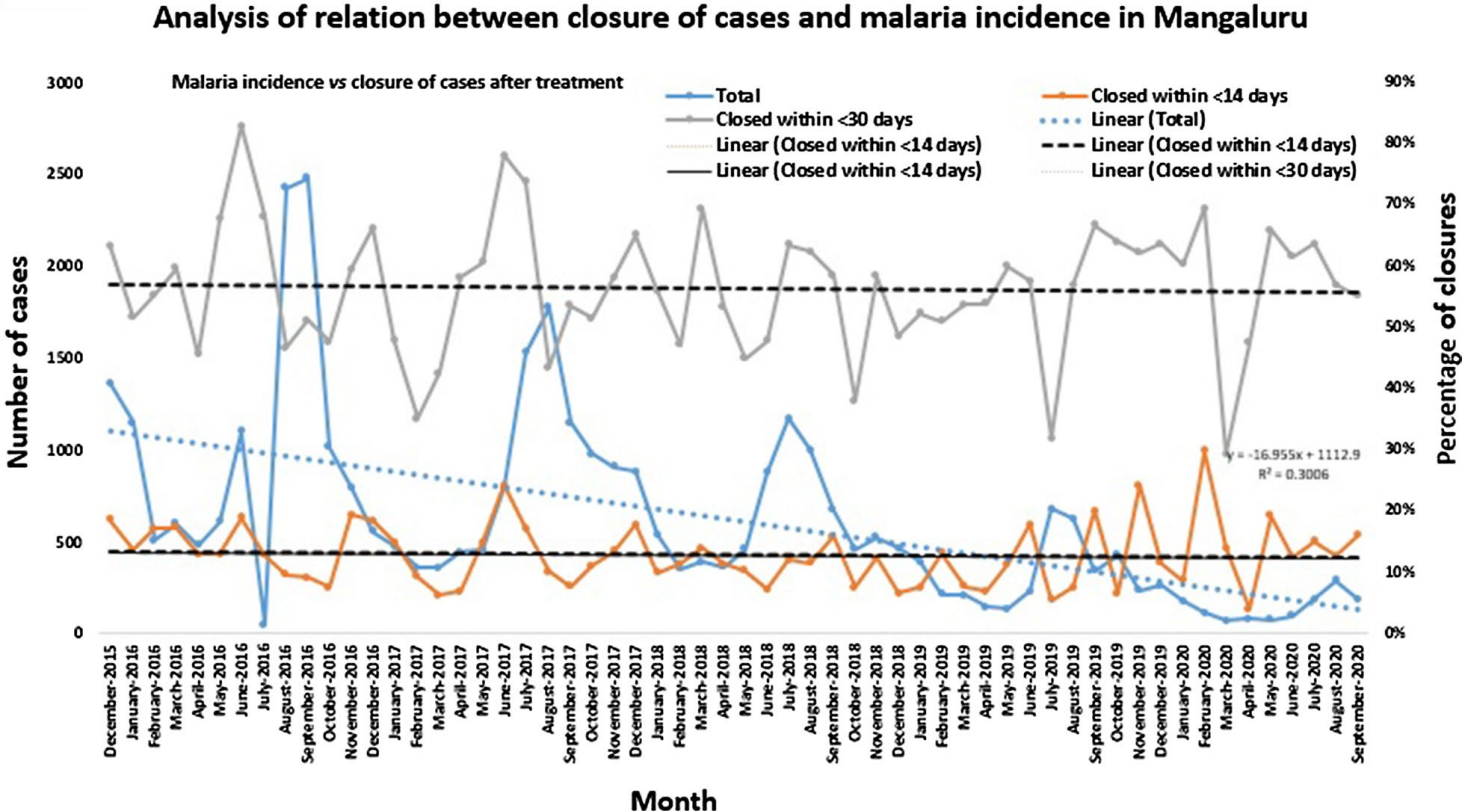
Analysis of relation between closure of cases and malaria incidence in Mangaluru

**Fig. 4:**
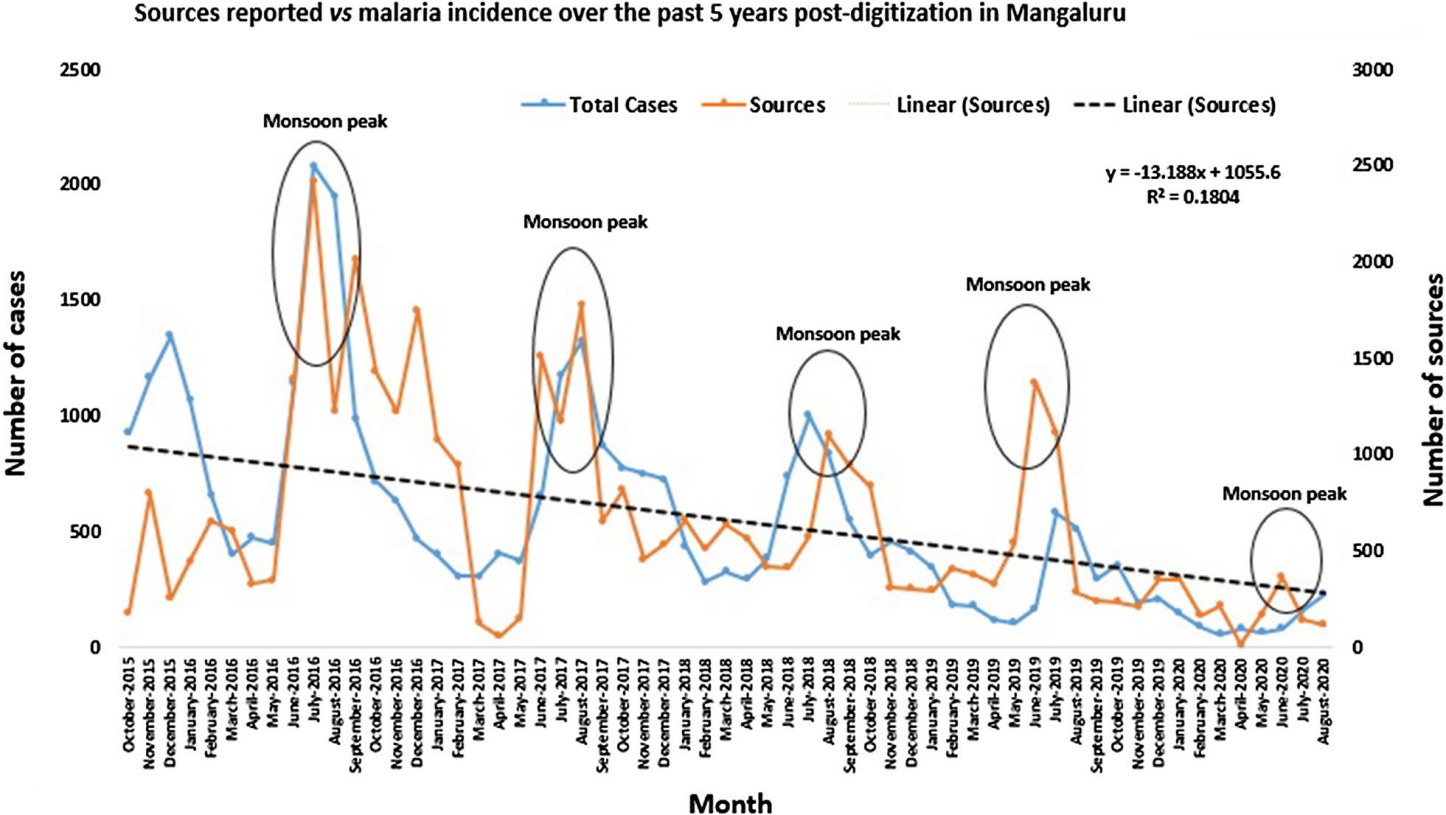
Sources reported *vs* malaria incidence over the past 5 years post-digitization in Mangaluru

Trends for the overall malaria incidence over 5 years is depicted in Fig. 1. The cases peaked during the monsoon season but an overall annual decreasing trend was observed. Improvement in reporting of cases from point-of- diagnosis on the web-based software is shown in Fig. 2. Most cases were reported on the same day or the next day. Similarly, Fig. 3 depicts the monthly malaria incidence as against the percentage of closure of cases within 14 and 30 days, respectively. The source of mosquito breeding habitats were identified in and around the residence of malaria patient. This activity was carried out soon after new cases were reported on the system. Source identification was highest during rainy monsoon and is further carried out during winter and summer periods. Details are given in Fig. 4.

**Fig. 5:**
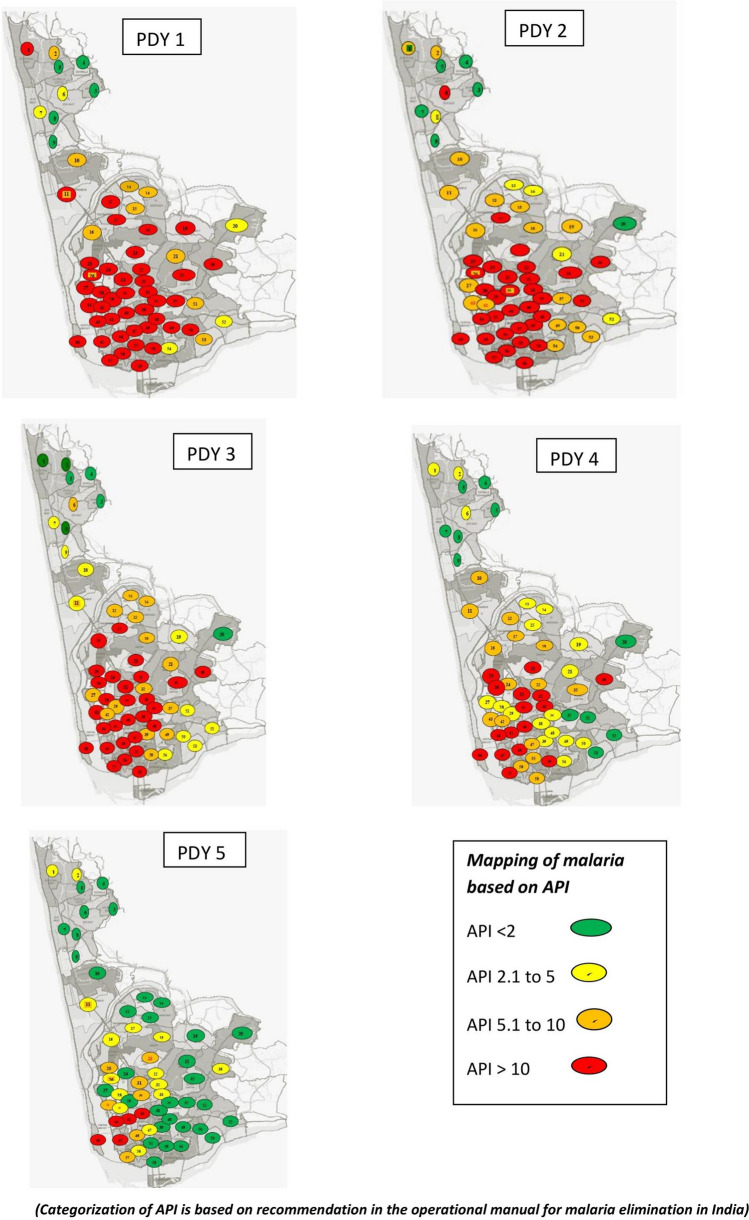
Map of Mangaluru with various wards depicting the areas based on API (cases per 1000 population) in PDY 1 through PDY 5

The ward-level depiction based on API from PDY 1 to PDY 5 is shown in Fig. 5. There was a gradual shrinking of malaria maps in the city. It can be noted that the wards with API in the red zone (API > 10) have reduced to only 5 wards in PDY 5 as against 43 prior to digitization. The wards in green (API ≤ 2) as well as yellow (API > 2.1 to 5) have increased over the years.

## Discussion

Mangaluru has been classified as a high-risk region for Urban Malaria by NVBDCP [11], endemic for malaria contributing to 85% of malaria cases in the state of Karnataka, India. Malaria being a dual host-disease estimation of *R*_*O*_ (reproduction number) is complex. Recent mathematical models have been used to estimate *R*_*O*_ which ranges from 1 to 3000 [12, 13]. Efficient participation of multiple stakeholders to manage both hosts and vectors determines the results of control measures. Failure to contain malaria over two decades, in spite of the ongoing control programme stipulated a new approach. MCS was introduced in October 2015 to improve ‘surveillance with timeline’ and dissemination of case details for appropriate action in the field [9]. Electronic surveillance system helps to connect all stakeholders with necessary information for expected time-bound response in the field to break the transmission chain. A multi-pronged, integrated approach involving all health care providers, time bound field responses i.e. active case detection and anti-mosquito measures in a geographical area is critical for containment and elimination of malaria thereafter.

MCS is a dedicated IT system which is also integrated to mobile technology, and is designed to be user friendly and easy to operate. However, it requires few months to train and implement the available functions of MCS by all the stakeholders namely in hospitals and diagnostic centers, and among field workers and administrators. Once offline data is captured in the device, synchronization with the system occurs whenever internet connectivity is available, and therefore, it can be used anywhere for data collection. Over 5 years there were consistent reversal of trends, and an overall reduction in malaria cases by 83%, while the monthly incidences reduced to double digits. The trend continued during COVID-19 pandemic when the entire health system was engaged in fighting against the disease. MCS affected most parameters for malaria and contributed to the effective reduction of cases in Mangaluru.

### Malariometric indices

Malariometric indices showed significant changes over 5 years. The incidence of both *Plasmodium vivax* and *Plasmodium falciparum* gradually decreased. Initially, ABER increased significantly with predominant contribution from passive surveillance. Stagnation between second and third year after implementing of MCS was a consequence of administrative decision to utilize MPWs for non-malarial work. In PDY 3, CMETs were formed to supplement the active surveillance and the results can be seen during PDY 4 and PDY 5. During COVID-19 pandemic, active surveillance could not be carried out efficiently resulting in decreased ABER and increased SPR. However, the incidence of malaria and API continued to decrease without any rebound increase in the ‘post-lockdown’ period. Thus, there is a need to have comprehensive approach for malaria elimination since it is a dual host disease with wide ranging *R*_*O*_ factor, dormant stage in humans and resistance to various strategies adopted for control or elimination. The ultimate goal of all strategies is to reduce API in the area and reduce the size of malaria map. A dedicated, user-friendly system which captures data with timeline will assist in micromanaging multiple strategies.

### Reporting of cases

Reporting of malaria cases was mandatory under the communicable disease act of 1969; notifiable disease act of Government of Karnataka, and Karnataka Private Medical Establishment Act 2007. Prior to the introduction of MCS, malaria cases were reported late by the private sector health institutions via email or never reported in spite of statutory requirements. Subsequent to the introduction of MCS, ‘smart surveillance’, training programmes were conducted for private hospitals. Monitoring and appraisal on quality of reporting system was carried out periodically. In the absence of newly diagnosed cases, hospitals were required to provide `zero malaria case’ report. With persistent motivation, behavioural changes were observed with respect to timely reporting of malaria cases by the diagnosticians, and it continued through PDY 5. Details of 89% of newly diagnosed cases were uploaded into the system within 48 h. Both public and private health care providers reported the malaria cases (Table 3). All these were passive case detection (PCD) from health facilities with exception of cases reported by ASARC and DVBDCO. Very high rate of passive case detection reflects ‘health-seeking behaviour’ of the population and is probably one of the reasons for decrease in incidence even during COVID-19 pandemic. Private sector contribution was higher than public health system and is an indication of definite compliance to non-reporting from private health system which was a major hurdle for malaria control in India [7]. As per World Health Organization, cases of malaria are reported only from public health care facilities, and hence a large number of cases are unreported thereby facilitating transmission [14, 15]. However, even where reporting rates in the public health sector are close to a 100% in some countries, more than 50% of malaria patients sought health care in the private sector [12]. Hence, reporting from private sector is crucial for malaria control.

Figure 2 indicates average time taken to report from the time of diagnosis. Capturing the case details and transferring this information to the health workers in the field is the key to initiate control activities. Robust reporting of PCD initiated active case detection (ACD), which is a very important factor for malaria elimination. It has been observed that early reporting from the diagnosticians continued even during COVID-19 epidemic thus resulting in disruption of transmission cycle.

### Field response within timeline

Efficient participation of multiple stakeholders is crucial for effective control measures. Multi-pronged, integrated approach is critical for containment of malaria and elimination thereafter. ‘Smart surveillance’ helped to connect all stakeholders with necessary information for anticipated response in the field to break the chain of transmission. ‘Time-bound’ field response i.e. active case detection and anti-mosquito measures in the geographical risk areas were carried out simultaneously. Immediate contact smears and identification of positive cases helped to reduce parasite pool available for transmission. Importance of time bound programme has been reported from China [16].

### Surveillance

In the initial year after MCS, an increase in incidence was documented suggesting improved surveillance. In subsequent years, there was a gradual reduction in incidence of malaria. This reduction was not uniform throughout the year. Although during and immediately after monsoon rains (June to October) there have been variable spikes in incidence, the number of cases gradually reduced during same year on year period (Fig. 1). Surveillance, early case detection, treatment and vector control measures were done as per the NVBDCP guidelines with variable results. With the introduction of MCS, the surveillance was robust time-bound and ‘incidence-centric’. Quick transfer of information from point-of-diagnosis to the field workers and surveillance thereafter contributed to 1.8% of reported cases of malaria in the city in PDY 5. Albeit small in number, it is of high significance for breaking the transmission cycle. Rapid reporting and information of geolocation have been the strength of malaria control system in Zanzibar and Swaziland [17–20]

### Mosquito control activities

All required field activities were recorded directly in the programme by MPWs using predesigned dropdown menu. This shift from manual documentation to MCS ensured appropriate field response including mosquito control measures by the MPWs. In the initial days after introduction of MCS the field movement of MPWs were monitored using GPS. Such monitoring and feedback brought about behavioural changes among health care providers in the preceding years. Transmission of malaria depends on *Ro* which in turn is determined by patient factors (PR or parasite ratio) and mosquito behaviour related to entomological inoculation rate (EIR) [21]. Therefore, it is imperative to prevent transmission of parasite from malaria patient to mosquito. An infected mosquito can continue to transmit sporozoites to many healthy individuals for a longer period. The risk of transmission to surrounding population can be minimized with anti-adult or anti-larval measures in houses around the residence of active malaria cases. Effective source reduction management happened over 5 years with gradual reduction of active breeding habitats (Fig. 4). Measures to reduce breeding and spread are important public health measures in malaria elimination operation [9].

### Local strategies

Eighteen months after digitization an administrative decision was taken to utilize the services of MPWs for non-malarial work resulting in diminishing efficiency in the field. Although the community visits increased by manifold during PDY 3, it was not translated to effective vector control measures and collection of smears as active surveillance reduced from 4.61 per incidence (PDY 2) to 2.8 per incidence (PDY 3). This resulted in a slump in the work and non-reduction of malaria incidence during PDY 3. A surge in the number of cases was observed in April–May 2017 which led to increase in malaria indices. To counter this inefficiency, CMETs were formed at district malaria unit in June 2018. The CMETs conducted ASARC along with anti-vector activities in the locality. Subsequent to CMETs surveillance, reduction of cases was observed in the fourth year. During PDY 5, because of COVID-19 pandemic, the entire nation was under lockdown, and the public health system was engaged in fighting this new disease. However, the CMETs continued carrying out the visits to malarial houses. This activity is probably the main reason for reduction of malaria cases during PDY 5.

The global effort of malaria control is in line with the ‘One World One Health’ concept, but then a globally defined ‘one-size-fits-all’ malaria control strategy would be inefficient in endemic areas [22, 23]. Introduction of MCS did aid in local modification of strategies. During analysis of new cases, clusters of new cases within a short period of one week, within a defined geographical area were identified (hot-spots) and strategically separate programmes were carried out. One such endeavour was targeted for labourers and daily wage earners. Generally, malaria clinics are open from 9 AM and to 5 PM, which were underutilized as it was not convenient for the manual labourers and daily wage earners and low socioeconomic class, as they were engaged in their income generation activities during that time. Hence, a mobile 24 × 7 clinic using a van and health care workers was introduced so that it could visit various places and could also be sent to the site if there was a phone call made to the central malaria helpline number. This helped in not only enhancing the diagnosis, but also treatment and prompt reporting of malaria in migrant population.

### Mapping and risk categorization

It has been a long-standing concern for epidemiologists to quantify and stratify risk for malaria. Risk categorization for strategies and programme management is the key to success of malaria eradication and elimination [21]. MCS captured data on real time basis for spatial risk classification. Geographical high-risk categorization is based on API, and 43 such wards recorded reduction of incidence by 80% and above over 5 years. Several wards converted from a high API red zone to a lesser API green or yellow zones (Fig. 3). Risk prediction model was applied for malaria elimination process [6, 20]. There is a role to understand geographic trends for planning the strategies at micro-level and further research and review are warranted. Moreover, it may be worthwhile to look at the socio-demographic characteristics of people in these areas as well as the activities like construction and migration or travel [22].

### COVID-19 pandemic and malaria control programme

In PDY 5, COVID-19 emerged as a major global public health challenge and disrupted malaria control programme in general. While February 2020 was mainly focused on preparation to plan strategies to control COVID-19, nationwide lockdown recorded a decrease in number of cases of all diseases as the hospitals were converted to Covid-19 facilities and care centres. Diversion of health care workforce towards COVID-19 management, total lockdown of the entire country, non-availability of transportation, closure or limited working hours of health facilities hampered anti-malarial activities for a short period of 5 to 6 months. However, active surveillance i.e. ASARC continued uninterrupted, routine house visits were reduced, but closure of cases continued quite effectively.

### Accountability

Strengthening of field work force and capacity building is essential in any public health programme. MCS did empower the field workers and it also helped in data-based micromanagement by the administrators as well as field workers. A bidirectional accountability was also observed i.e. from field force to administration and *vice-versa*. ASARC, time bound action in the geographical area surrounding the new malarial case, continuity in control measures especially during low transmission period (non-monsoon period). The necessity of closing the case on day 14, and its measure reflects functional accountability by the field work force. Closure of cases steadily increased and contributed to reduction of malaria incidence. There were delays in closure of cases as a result of multiple factors, like non-working days, non-availability of the patient upon visit to home, migration, etc. Nevertheless, over 90% cases were tracked and closed subsequently. An inverse relation between closure and malarial incidence was observed (Fig. 2). Hence, the function of ‘close a case’ ascertained complete treatment and parasite clearance thereby contributing to transmission control.

### Future scope

The five-year data indicated that technology has a major role to play in evaluating epidemiology of malaria as well as malariogenic factors. Learning from MCS application should help to upgrade functions, incorporate analytical and predictive output, warning and alarm systems for compliance in the field. Ideally, there is a need to design IT system driven field response for both treatment and vector control analytics and predictions. Since most control measures are similar for all vector borne diseases they should be brought under the purview of independent system to manage vector borne diseases or even other infectious diseases.

**Fig. 6:**
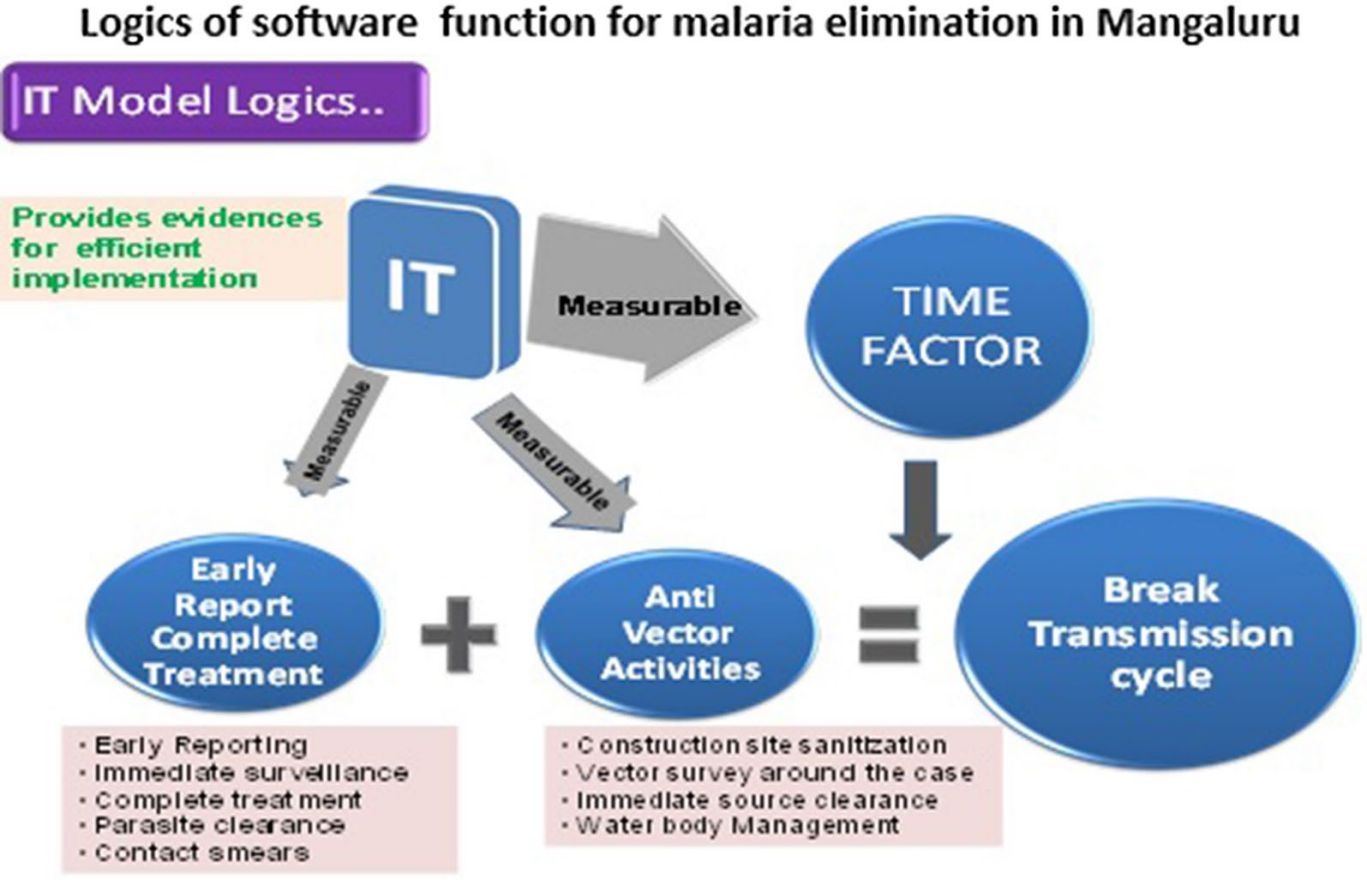
Logics of software function for malaria elimination in Mangaluru

Malaria elimination is being envisaged by 2030 in India. An excellent information system should be at the core of malaria elimination programmes to ensure that all cases are detected and responded to an effective and timely manner. Investment in robust, response-focused systems is essential to achieve malaria elimination. The operational manual elaborates the strategies. However, these strategies need to be structured with ‘time-bound’ interventions. Figure 6 provides functional description of MCS for good micromanagement which is essential for malaria elimination [5, 6]. All micromanagement data regarding treatment and vector control measures can be quantified in relation to ‘time frames’ for each action. Transmission cycle is broken effectively if field interventions are carried out in the first 7 days of diagnosis. Transmission occurs locally around a reported case, and it is logical to implement effective vector control activity and measure this activity simultaneously. A standardized surveillance system landscaping was conducted in 16 countries between 2015 and 2017 in collaboration with governmental malaria programmes. The landscaping analysis identified multiple gaps in current malaria surveillance systems [24]. Nema et al. also pointed out these gaps [25], and suggested for a robust digital health care service in India [26]. Smart surveillance is able to measure and micromanage control measures for designing local strategies.

## Conclusion

Surveillance is the backbone of an effective system to support malaria elimination. Poor surveillance data will prevent countries from monitoring of progress towards elimination process. MCS driven reporting, field responses and creation of big data are effective tools to improve malaria control operations. MCS helped to achieve (a) robust reporting of cases from all health sectors; (b) incident-centric active surveillance; (c) complete treatment with documentation of parasite clearance; (d) targeted mosquito control measures; (e) sustained field activities though both high and low transmission periods; (f) modify strategies for local control of both disease and vector(s). IT system brought about behavioural changes among health care providers and community. Information systems like MCS are essential to maintain control and continuity, even when the civic body is compelled to divert resources and fight new battles. It is clear from the five-year data that this method of `smart surveillance’ is reproducible with minimum training and also improves human resource micromanagement.

## Data Availability

The data used in this study are archived with Dr BS Baliga and available from them upon reasonable request.

## Abbreviations

TABs: Tablets
GIS: Geographic information system
GPS: Geographical positioning system
NVBDCP: National Vector Borne Disease Control Programme
MPWs: Multipurpose workers
MCS: Malaria control system
MIS: Malaria information system
CMETs: Comprehensive malaria elimination teams
DY: Digitization year
PDY: Post-digitization year
ASARC: Active surveillance around reported case
ABER: Annual blood examination rate
API: Annual parasite incidence
SPR: Slide positivity rate
SFR: Slide falciparum rate
PCD: Passive case detection
ACD: Active case detection
IT: Information Technology
MCC: Mangaluru City Corporation
